# Supplementary material for: Theory of optical axion electrodynamics and application to the Kerr effect in topological antiferromagnets
Source: Nat Commun. 2022 Dec 9;13:7615. doi: 10.1038/s41467-022-35248-8 (PMC9734152; doi:10.1038/s41467-022-35248-8)
Supplement: Supplementary file 1 — Supplementary Information [file 41467_2022_35248_MOESM1_ESM.pdf]

Supplementary Information for "Theory of optical axion electrodynamics and application to the Kerr effect in topological antiferromagnets"

Junyeong Ahn,[1,*] Su-Yang Xu,[2,†] and Ashvin Vishwanath[1,‡]

[1]*Department of Physics, Harvard University, Cambridge, MA 02138, USA*

[2]*Department of Chemistry and Chemical Biology, Harvard University, Cambridge, MA 02138, USA*

(Dated: November 18, 2022)

* junyeongahn@fas.harvard.edu

† suyangxu@fas.harvard.edu

‡ avishwanath@g.harvard.edu

Supplementary Note 1. Gyrotropic birefringence and natural optical activity

Here we review the theoretical analysis of gyrotropic birefringence and natural optical activity. This is an analysis of the bulk current response in contrast to the surface current response that we present in the main text. Therefore, axion electrodyamics is not described by the approach in this section [1]. Below we mainly follow the work of Malashevich and Souza [1]. Along the way, we compare our notations with the notations in that paper [see Eqs. (6) and (9)].

We consider a three-dimensional homogeneous system without boundaries. By the multipole expansion up to electric-quadrupole/magnetic-dipole order, the bulk current density is written as

$$\begin{aligned} J_i &= \dot{P}_i - \frac{1}{2}\sum_j \partial_j \dot{Q}_{ij} + \sum_{jk} \epsilon_{ijk}\partial_j m_k \\ &= -i\omega\tilde{\alpha}_{ij}E_j - i\left[i\sum_l(\tilde{G}_{il}\epsilon_{ljk} + \epsilon_{ikl}\tilde{\mathfrak{G}}_{lj}) + \frac{\omega}{2}(\tilde{a}_{ijk} - \tilde{\mathfrak{a}}_{ikj})\right]\partial_k E_j \\ &= \sum_j \sigma_{ij}E_j - i\sum_{j,k}\sigma_{ijk}\partial_k E_j, \end{aligned} \tag{1}$$

where we use that induced multipole moments are

$$\begin{aligned} P_i &= \tilde{\chi}_{ij}E_j + \frac{1}{2}\tilde{a}_{ijk}\nabla_k E_j + \tilde{G}_{ij}B_j \\ Q_{ij} &= \tilde{\mathfrak{a}}_{ijk}E_k \\ M_i &= \tilde{\mathfrak{G}}_{ij}E_j \end{aligned} \tag{2}$$

and that response functions do not change spatially. We are interested in the first order in momentum.

$$\sigma_{ijk} = i\sum_l(\tilde{G}_{il}\epsilon_{ljk} + \epsilon_{ikl}\tilde{\mathfrak{G}}_{lj}) + \frac{\omega}{2}(\tilde{a}_{ijk} - \tilde{\mathfrak{a}}_{ikj}) \tag{3}$$

After symmetrizing the first two indices, we have

$$\begin{aligned} \sigma^S_{ijk} &= \frac{1}{2}\left(\sigma_{ijk} + \sigma_{jik}\right) \\ &= i\sum_l(\alpha_{il}\epsilon_{ljk} + \epsilon_{ikl}\alpha_{jl}) + \omega\gamma_{ijk}, \end{aligned} \tag{4}$$

and, similarly, we have the anti-symmetrized part

$$\begin{aligned} \sigma^A_{ijk} &= \frac{1}{2}\left(\sigma_{ijk} - \sigma_{jik}\right) \\ &= i\sum_l(\beta_{il}\epsilon_{ljk} - \epsilon_{ikl}\beta_{jl}) + \omega\xi_{ijk}, \end{aligned} \tag{5}$$

where we define

$$\begin{aligned}
\alpha_{ij} &= G_{ij}, \\
\beta_{ij} &= -iG'_{ij}, \\
\gamma_{ijk} &= -\frac{i}{2}(a'_{ijk} + a'_{jik}), \\
\xi_{ijk} &= \frac{1}{2}(a_{ijk} - a_{jik}),
\end{aligned} \tag{6}$$

and

$$\begin{aligned}
a_{ijk} &= \frac{\tilde{a}_{ijk} + \tilde{\mathfrak{a}}_{jki}}{2}, \\
a'_{ijk} &= i\frac{\tilde{a}_{ijk} - \tilde{\mathfrak{a}}_{jki}}{2}, \\
G_{ij} &= \frac{\tilde{G}_{ij} + \tilde{\mathfrak{G}}_{ji}}{2}, \\
G'_{ij} &= i\frac{\tilde{G}_{ij} - \tilde{\mathfrak{G}}_{ji}}{2}.
\end{aligned} \tag{7}$$

The conductivity tensor is related to the origin-independent combinations of the magneto-electric coupling and electric quadrupole susceptibility by

$$\begin{aligned}
\sigma^S_{ijk} &= i\sum_l (\tilde{\alpha}_{il}\epsilon_{ljk} + \epsilon_{ikl}\tilde{\alpha}_{jl}) + \omega\tilde{\gamma}_{ijk}, \\
\sigma^A_{ijk} &= i\sum_l (\tilde{\beta}_{il}\epsilon_{ljk} - \epsilon_{ikl}\tilde{\beta}_{jl}),
\end{aligned} \tag{8}$$

where

$$\begin{aligned}
\tilde{\alpha}_{ij} &= \frac{1}{3i}\sum_l \epsilon_{jkl}\sigma^S_{ikl} = \alpha_{ij} - \frac{1}{3}\delta_{ij}\sum_k \alpha_{kk} + \frac{\omega}{3i}\sum_{k,l}\epsilon_{jkl}\gamma_{ikl} = T_{ij}, \\
\tilde{\gamma}_{ijk} &= \frac{1}{3\omega}(\sigma^S_{ijk} + \sigma^S_{jki} + \sigma^S_{kij}) = \frac{1}{3\omega}(\gamma_{ijk} + \gamma_{jki} + \gamma_{kij}) = -iS_{ijk}, \\
\tilde{\beta}_{ij} &= \frac{1}{4i}\sum_{k,l}\epsilon_{jkl}(2\sigma^A_{ikl} - \sigma^A_{kli}) = \beta_{ij} + \frac{1}{4i}\sum_{k,l}\epsilon_{jkl}(2\xi_{ikl} - \xi_{kli}) = -iT'_{ij},
\end{aligned} \tag{9}$$

and T_{ij}, T'_{ij} and S_{ijk} are the notations we use in the main text. Note that only the traceless part of the magneto-electric coupling appears in the bulk conductivity tensor σ_{ijk}, indiciating that the axion magneto-electric effect is missing in this approach.

---

[1] Malashevich, A. & Souza, I. Band theory of spatial dispersion in magnetoelectrics. *Phys. Rev. B* **82**, 245118 (2010).

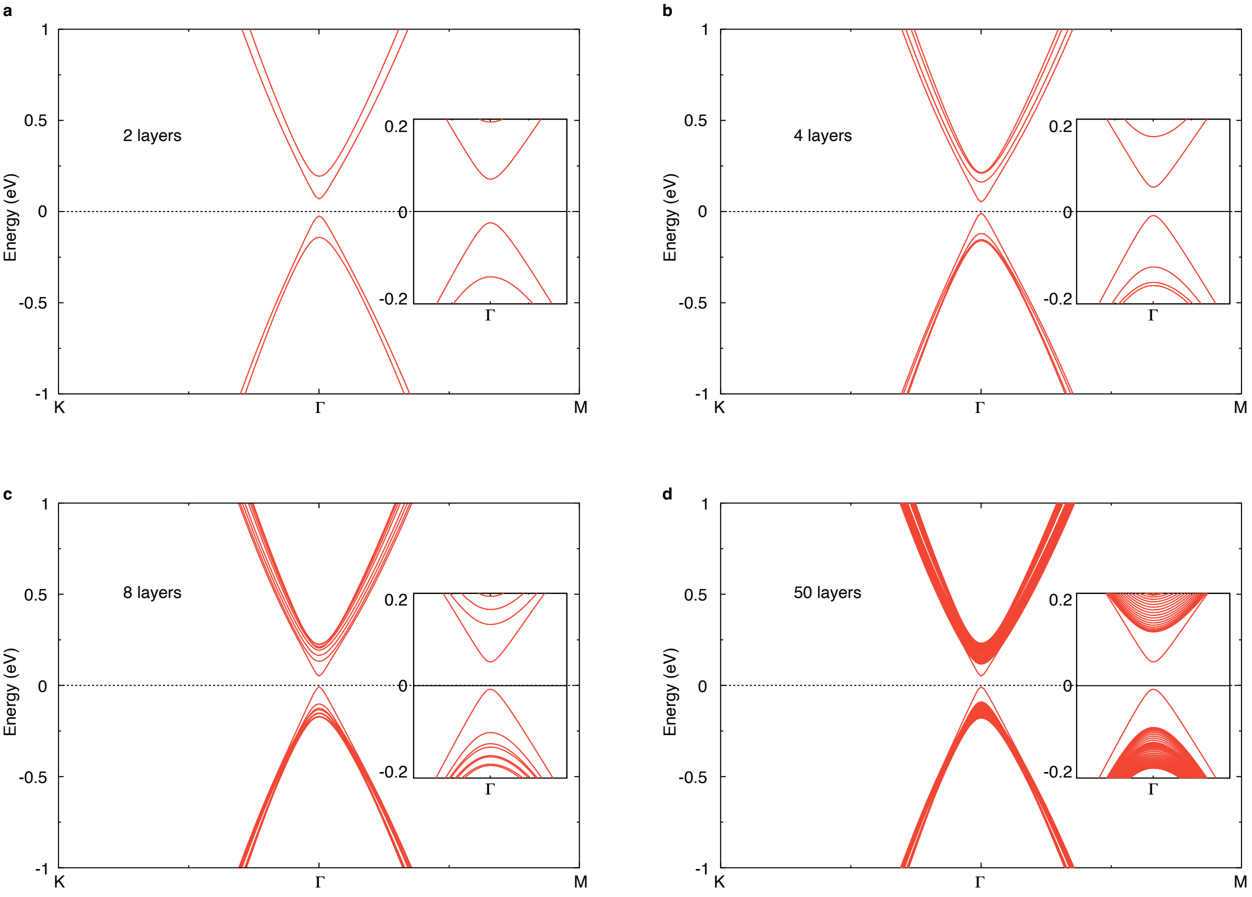

Supplementary Fig. 1: Layer dependence of the band structure.

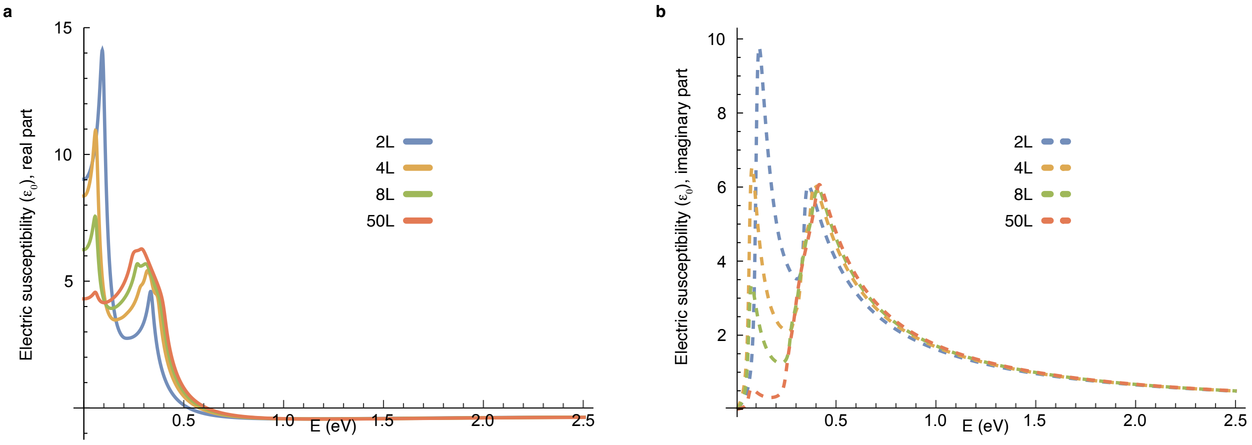

Supplementary Fig. 2: Layer-dependent spectrum of the electric susceptibility χ_{xx}**.** Two peaks in the spectrum correspond to the surface and bulk band gaps.
